# Assessing motivation and readiness to change for weight management and control: an in-depth evaluation of three sets of instruments

**DOI:** 10.3389/fpsyg.2015.00511

**Published:** 2015-05-11

**Authors:** Martina Ceccarini, Maria Borrello, Giada Pietrabissa, Gian Mauro Manzoni, Gianluca Castelnuovo

**Affiliations:** ^1^Istituto Auxologico Italiano IRCCS – Ospedale San GiuseppeVerbania, Italy; ^2^Psychology Department, University of BergamoItaly; ^3^Psychology Department, Catholic University of MilanMilan, Italy

**Keywords:** motivation, readiness to change, weight-management, Transtheoretical Model, assessment, obesity, overweight

## Abstract

It is highly recommended to promptly assess motivation and readiness to change (RTC) in individuals who wish to achieve significant lifestyle behavior changes in order to improve their health, overall quality of life, and well-being. In particular, motivation should be assessed for those who face the difficult task to maintain weight, which implies a double challenge: weight loss initially and its management subsequently. In fact, weight-control may be as problematic as smoking or drugs-taking cessation, since they all share the commonality of being highly refractory to change. This paper will examine three well-established tools following the Transtheoretical Model, specifically assessing RTC in weight management: the University of Rhode Island Change Assessment Scale, the S-Weight and the P-Weight and the Decisional Balance Inventory. Though their strengths and weaknesses may appear to be rather homogeneous and similar, the S-Weight and P-Weight are more efficient in assessing RTC in weight management and control. Assessing motivation and RTC may be a crucial step in promptly identifying psychological obstacles or resistance toward weight-management in overweight or obese hospitalized individuals, and it may contribute to provide a more effective weight-control treatment intervention.

## Introduction

Morbid obesity and overweight are a widespread epidemic (‘Globesity’) reported in both industrialized as well as developing countries (Wadden et al., 2002). There is a consensus among health professionals and scientists about the existence of environmental factors favoring the rise of this global epidemic (World Health Organization [WHO], 2014). Such features influence the compatibility between what is offered nutrition-wise by the socio-psycho-economic living context, and what is biologically needed by the human body to reach an optimal functionality and to survive (Toft et al., 2007). Healthy nutrition and regular physical activity are considered essential protective factors in health promotion and risk prevention of chronic diseases (Britt et al., 2004). Nonetheless, the sole knowledge of healthy nutrition standards and of healthy dietary prescriptions and of regular physical activity programs is not enough to achieve optimum lifestyle, or to reduce excessive food-consumption and bodyweight (Resnicow et al., 2008).

According to Bautista-Castaño et al. (2004), the majority of obese and overweight individuals do not continue weight-loss programs, and only a few of them who do so, actually lose weight. Empirical research demonstrated that the majority of obese people, who managed to lose weight during hospital-based interventions, go back to their original weight in three to 5 years after treatment (Castelnuovo et al., 2010). In other words, the problem is not to start a diet but to continue it, avoiding regaining the previously lost weight and slipping into a vicious cycle (Tremblay and Sánchez, 2012). In this respect, readiness to change (RTC) seems to be one of the most promising factors promoting behavior change in individuals who need to modify their lifestyle for health purposes. Personal motivation can, in fact, dramatically influence treatment adherence and effectiveness as well as the choice of intervention (Resnicow et al., 2008). Several studies have pointed out that motivational techniques encourage weight-loss by favoring adherence to weight-loss and weight management programs, with positive results (Ryan and Deci, 2000; Wilson and Schlam, 2004; Pietrabissa et al., 2012). This has also been highlighted by Salvini et al. (2012) who pointed out how Miller and Rollnick’s (1991) motivational interview (MI) and the Self-Determination Theory (SDT, by Ryan and Deci, 2000), evidence that motivational dynamics can be resourceful elements helping individuals become proactive participants in their behavior change.

The vast majority of motivational measures adopted in the clinical setting have been developed for substance-addiction (Di Clemente and Prochaska, 1982, 1985; Prochaska and Di Clemente, 1983, 1984, 1986; Prochaska et al., 1992a; Velicer et al., 1995) and generally relate to Prochaska and Di Clemente’s Transtheoretical Model (TTM) based on RTC and on the Stages of Change (SOC; Prochaska and Di Clemente, 1984; Prochaska et al., 1994, 2008; Prochaska and Velicer, 1997; Prochaska and Norcross, 2006). The TTM offers a comprehensive theoretical framework determining RTC and promoting tailored interventions according to the patient’s motivation in weight management (Prochaska et al., 1992b), also preventing dropouts (Rossi et al., 1995; Johnson et al., 2008). This model defines the relationships between the stages and the processes of change, decisional balance (DB; i.e., advantages and disadvantages of behavior change), self-efficacy, and relapse (Prochaska and Di Clemente, 1986). The SOC of the TTM are: Precontemplation, Contemplation, Preparation, Action, and Maintenance, in which individuals move forward and at times backward, re-starting the cycle. Though individuals may go back to a stage they had already been through, previous experiences still constitute a useful step for behavior change. This is the reason why the model is best depicted as a spiral (Prochaska and Di Clemente, 1983; Di Clemente and Prochaska, 1985; Prochaska et al., 1992a).

During the initial Precontemplation stage, individuals are not willing to change within the next 6 months. While some could totally refuse the change, others may wish to achieve it at some point in the future, though not within the next 6 months. During the Contemplation stage, individuals are thinking about changing their target-behavior within the next 6 months and are keen to receive information on their problem. The Preparation stage is characterized by a commitment toward the change, possibly within the next months. Generally, individuals at this phase have already tried changing their dysfunctional behavior before, or they have been making efforts to prepare for change. In the Action stage individuals have put into practice their attempts to change, and actually operated this modification within the past 6 months. At this level, the risk of relapsing is rather high, since individuals are engaged in something totally new. Hence, they need to pay a great deal of attention in order to avoid falling straight back into their old unhealthy lifestyles. In the final stage, Maintenance, individuals have actually changed their problematic behavior for at least 6 months. At this level, the change has become part of their life and they are less likely to relapse than in the previous stages, although relapse prevention is still advisable (Prochaska and Velicer, 1997; Redding et al., 2000).

Assessing RTC usually coincides with classifying an individual at a given stage to identify his/her level of the problem awareness (reasons for change), his/her willingness to change (commitment for change), and his/her actions for change. Questionnaires assessing TTM constructs in weight management are frequently adapted from other tools designed to measure RTC in addictive disorders (Greene and Rossi, 1998; Marshall and Biddle, 2001). In the context of weight management, the assessment of RTC and SOC has generally been carried out with separate evaluations of dietary behavior change and physical exercise. According to Horwath (1999), using specific measures of both dietary behavior and exercise can contribute to provide a reliable and efficient assessment in weight-management, according to the TTM constructs.

Many clinician-rated and patient-rated instruments have been developed to measure RTC in the clinical setting during the last 20 years. However, a motivational assessment in obese hospitalized in-patients does not always correspond to an early, accurate use of suitable tools. This paper aims at evaluating three major assessment instruments, the University of Rhode Island Change Assessment Scale (URICA; McConnaughy et al., 1983, 1989; Rossi et al., 1995), the S-Weight/P-Weight (Andrés et al., 2011), and the Decisional Balance Inventory (DBI; O’Connell and Velicer, 1988). This work intends to provide an in-depth comparison between these tools, identifying which one is more suitable in detecting RTC in weight-management within obese hospitalized in-patients.

The selection of the three measures was based upon specificity regarding SOC, processes of change and motivation in weight management and more generally, the decision of whether or not to maintain weight according to the SOC of the TTM model. Thus, strengths and weakness of these three sets of questionnaires analyzed in this brief review will be pointed out and the most recommendable tool will be clearly identified. It is important to verify which instrument is more useful to evaluate motivational levels in weight-management amongst obese or overweight individuals. This is because an efficient appraisal in this context could be fundamental in later providing patients with the best possible psychological support and most suitable weight-loss treatment.

## The Assessment of Readiness to Change in Weight Management and Weight Control

The URICA is the most widely studied measure of readiness for change designed for an adult target population. The questionnaire was originally used for patients in psychotherapy reporting on their specific problem in treatment (McConnaughy et al., 1989). However, the measure can be applied to assess the respondent’s RTC on a range of different problems such as addictions, smoking cessation, alcohol, and cocaine use (Rossi et al., 1995). The URICA has been successfully used with other problematic behaviors including obesity, diet and weight management (Prochaska et al., 1992b). The scale is a 32-item self-report measure that includes four subscales measuring Prochaska and Di Clemente’s TTM SOC: Precontemplation, Contemplation, Action, and Maintenance (Prochaska and Velicer, 1997). Responses are given on a 5-point Likert scale ranging from 1 (strong disagreement) to 5 (strong agreement). The test contains eight items for each of the subscales. The latter can be combined arithmetically (by summing up scores on the Contemplation, Action, and Maintenance subscales and by subtracting the score on the Precontemplation subscale) to yield a second-order continuous RTC score assessing RTC at treatment entrance (Prochaska et al., 1994; Greene et al., 1999).

The URICA is designed to be a continuous measure; subjects can obtain high scores on more than one of the four stages. Higher total scores indicate a greater RTC. For each subscale, individuals can score from a minimum of 1 to a maximum of 40 and a total test score ranging from a minimum of 1 to a maximum of 160 can be obtained. An overall low RTC level corresponds to a total score below 80, while a high RTC is represented by total scores above 80 (McConnaughy et al., 1989; Andrés et al., 2011). The URICA considers the transition between the SOC as being a gradual progression rather than a discontinuous and casual movement (McConnaughy et al., 1983). In the clinical setting, professionals may utilize this instrument to evaluate an individual’s motivation for change level and use this material to monitor and carry out specific and personalized treatment interventions. In fact, the four subscales scores of the test can be used to delineate modifications in attitudes related to the target-behavior, according to the individual’s specific SOC (McConnaughy et al., 1989; Andrés et al., 2011).

The URICA has good internal consistency with coefficient alphas ranging from 0.79 to 0.89 across the four subscales, even in follow-up studies (Andrés et al., 2011). Good reliability, construct validity and psychometric properties of the URICA have been established for a range of behavioral conditions (Willoughby and Edens, 1996; Pantalon et al., 2002; Dozois et al., 2004; Henderson et al., 2004). The construct validity of the URICA has been supported through factor and cluster analyses demonstrating that the SOC are associated with different behavioral profiles (McConnaughy et al., 1989). The latter reflect the possibility that respondents are likely to engage in actions and behavior representing more than one stage at a time. Moreover, the correlations between the questionnaire subscales suggest that adjacent stages are more strictly linked than non-adjacent ones (Rossi et al., 1995). There is consistent evidence supporting the transtheoretical four-factor structure of the URICA given by both principal component analysis (PCA) and structural equation modeling (McConnaughy et al., 1989; Andrés et al., 2011). Furthermore, the measure showed a good predictive validity, in foreseeing attendance and weight-loss in a study carried out by Prochaska et al. (1992b). In fact, the authors found that attendance was significantly predicted by higher scores on the Action subscale and by lower Precontemplation and Maintenance subscales scores.

Additionally, the URICA can also be used to measure processes and outcome variables for a range of health and addictive behaviors. Nonetheless, because the relationships among subscales shift as individuals move into Action and Maintenance, particular attention should be paid in assessing changes in pre-post design studies (Prochaska et al., 1992a). The questionnaire is very easy to administer since it only requires 5–10 min for completion and it can also be self-administered. Furthermore, no specific training is required for administration and the scoring can be carried out by any staff member by hand, in 5–10 min. The authors particularly recommend applying the questionnaire to evaluate progress during treatment and the outcome of specific interventions such as weight-loss and weight-maintenance (Andrés et al., 2011). In fact, the tool demonstrated to be reliable in a study on 184 hospital staff members engaged in a 10-week treatment program for weight-control (Prochaska et al., 1992b). However, the structure and internal consistency of the scale on weight-control samples is still somewhat unclear. Moreover, the questionnaire scale scores relate to simple unit weighting of items and thus involve either a total score or a mean scale score, which may require cluster analysis and standardized scoring if used in large sample sizes (Prochaska and Norcross, 2006).

The S-Weight and P-Weight are two self-report questionnaires respectively investigating the SOC and the processes of change defined by Prochaska and Di Clemente (1985). The S-Weight consists of five mutually exclusive items; respondents are asked to choose one of the five SOC to be allocated to among Precontemplation, Contemplation, Preparation, Action, and Maintenance (Di Clemente et al., 1991). The S-Weight is designed to measure the SOC as applied to weight management asking respondents to choose the answer that best corresponds to their current weight-loss situation (Andrés et al., 2009). The P-Weight consists of 34 items measuring individuals’ readiness to engage in a diet and in physical activity. The questionnaire is based on the hypothesised processes which individuals use across the SOC in order to manage their body weight (Andrés et al., 2011).

On the P-Weight, answers are given on a five-point Likert scale ranging from 1 (strong disagreement) to 5 (strong agreement). The four processes of change measured by the P-Weight that are implicated in weight management are: Emotional Re-evaluation (EmR), Weight Management Actions (WMA), Environmental Restructuring (EnR), and Weight Consequences Evaluation (WCE). The EmR process scale is comprised of 13 items, the WMA process scale is assessed by seven items, and the WCE process scale consists of nine items while the EnR process scale is evaluated by five items. Scores for each of the four processes of change can be calculated by summing up the scores obtained on items belonging to the same subscale. None of the items are reverse scored (Andrés et al., 2011). The measurement structure has four freely correlated first-order factors as revealed by the PCA and the confirmatory factor analysis (CFA). Four scores corresponding to the four processes of change can therefore be obtained from this questionnaire. However, the scores from the different subscales should be transformed onto a scale from 0 to 100 (from a minimum of 0 reflecting no use of a given process of change to a maximum of 100 being full-use of that process), in order to be comparable with one another. A higher use of a process is represented by scores above 50 (Andrés et al., 2011).

The P-Weight has good internal consistency with Cronbach’s alpha coefficients ranging from 0.781 to 0.938 for the different factors, while it has excellent internal consistency when considering the whole scale (the total scale Cronbach’s alpha is 0.960). Corrected item-total correlations of the measure are also adequate, ranging from 0.322 to 0.865 (Andrés et al., 2011). Moreover, the questionnaire has satisfactory convergent validity with the ‘drive for thinness’ subscale of the EDI-3 and the ‘diet’ subscale of the EAT-40. The four subscales of the P-Weight positively correlate with other scales measuring concern with dieting. This has been demonstrated by the original validation study conducted on 556 University students and on 167 overweight and obese patients enrolled in a hospital-based weight management program (Andrés et al., 2011).

The S-Weight and the P-Weight questionnaires are thus able to assess the relationship between stages and processes of change in weight-management. In this respect, the two measures allow identifying which processes of change individuals use the most according to the stage of change they are in, for what concerns weight-control (Andrés et al., 2011). The P-Weight questionnaire is very easy to administer since it requires less than 10 min for completion and it can be self-administered. No trained staff is necessary for administration and the scoring can be carried out by hand, in around 15 min time. However, in order to obtain the processes of change scores (from the P-Weight), across the SOC (from the S-Weight), more complex data analytic processes are needed, and the use of a statistical software is advisable.

The DBI was designed to evaluate decision making for weight-control considering two main dimensions, the pros and cons of losing weight (O’Connell and Velicer, 1988). Within the context of the TTM, the pros and cons demonstrate a close association between decision-making and an individual’s stage of change. Part of the decision to move from one stage to the next is based on the relative weight given to the pros and cons of changing the target-behavior. In fact, the comparative weighing of the pros and cons varies depending on the individual’s stage of change (Prochaska et al., 1994). Factor analytic studies (Prochaska et al., 1994, p. 27, 28; Andrés et al., 2011) have consistently supported the importance of the pros and cons of behavior change and the relationship between the SOC and DB (O’Connell and Velicer, 1988).

The DBI is a self-report measure consisting of two constructs addressing cognitive and motivational aspects of human decision-making based on Janis and Mann’s (1977) decision making model (O’Connell and Velicer, 1988, p. 46) and the TTM theoretical framework (Prochaska and Di Clemente, 1982). The test evaluates motivation with two main subscales, namely the ‘pros’ and the ‘cons’ of change. The two-factor structure of the test has been confirmed by PCA (Prochaska et al., 1994). The DBI has 20 items (10 items per subscale), asking to rate the importance of each statement in influencing the respondent’s decision on whether or not to lose weight. Answers are given on a five-point Likert Scale ranging from 1 (Not important at all), to 5 (Extremely important), and the questionnaire completion only takes about 5 min model (O’Connell and Velicer, 1988). The scoring is very simple and straightforward: by summing up the scores obtained from all even numbered questions it is possible to gain the score of the Pros scale while by summing up the scores obtained from all odd numbered questions a score for the Cons scale is attained. Thus, the range of possible total scores on each subscale ranges from a minimum of 10 to a maximum of 50. A total DB score is calculated by subtracting the cons score from the pros one. A higher DB score is associated with a greater motivation and RTC in weight-management. No trained staff is required neither for scoring nor for administration (O’Connell and Velicer, 1988).

The DBI has very good internal consistency with Cronbach’s alpha coefficients of 0.91 and 0.84, respectively, for the pros and cons scales. The measure construct validity was assessed by the validation study on 264 college students (O’Connell and Velicer, 1988). Findings revealed that the cons of weight-loss were significantly higher than the pros in the precontemplation stage, while in the contemplation, action and maintenance stages the trend was reversed. Thus, there is a clear pattern of relationships between weight-loss pros and cons and the TTM SOC. Such results were also found in several other studies on various health behavior problems (Prochaska et al., 1994; Redding et al., 2000). Up to date, the most recent validation of the DBI has been carried out in a study on the pros and cons of reducing dietary fat consumption in a large sample of adolescents (Rossi et al., 2001). However, more research on the psychometric properties of the DBI within the clinical setting and on adults’ weight-control samples is needed.

## Readiness to Change in Weight Management and Weight Control Instruments: Implications for Research and Clinical Practice

Among the tools analyzed throughout this paper, the URICA, the S-Weight and the P-Weight and the DBI, some measures appear to be more advantageous within the weight-management context. In fact, the S-Weight and the P-Weight, as shown in the summary provided in **Table 1**, seem to be the most consistent tools in assessing RTC in weight-control. Though the instruments described earlier may appear to be homogeneous in their strengths and weaknesses, the S-Weight and the P-Weight are more efficient toward a sound RTC assessment for what concerns weight-control, for several reasons. All three sets of questionnaires are appreciable given their short time completion and the unnecessary presence of trained personnel for administration. However, the S-Weight and the P-Weight are the only questionnaires among the abovementioned which at present, have been fully validated on overweight and obese in-patients. Moreover, these instruments seem to adequately and accurately detect specific RTC associated with weight-management, by measuring both processes of change and SOC according to the TTM (Andrés et al., 2011).

**Table 1:** Measurements characteristics.

Measure name	Validation study	No. of items	Tool characteristics	Advantages	Disadvantages
S-Weight and P-Weight	Andrés et al. (2011), “The Transtheoretical Model (TTM) in Weight Management: validation of the Processes of Change Questionnaire”, Obes Facts; 4:433–442 Andrés et al. (2009), “Establishing the stages and processes of change for weight loss by consensus of experts,” Obesity; 17:1717–1723.	S-Weight: five items, P-Weight: 34 items	Self-report measures assessing the stages and the processes of change. The S-Weight has five mutually exclusive items each representing the five stages of change (SOC; Precontemplation, Contemplation, Preparation, Action and Maintenance) in weight-management. The P-Weight has 34 items measuring four processes of change: emotional re-evaluation (EmR), Weight Management Actions (WMA), Environmental Restructuring (EnR) and Weight Consequences Evaluation (WCE). Responses on a 5-point Likert scale ranging from 1 (strong disagreement) to 5 (strong agreement). Item distribution is uneven: 13 items score for EmR, seven items score for WMA, nine items score for WCE, and five items score for EnR. Subscales scores are calculated by summing up scores obtained on items belonging to the same subscale. Each subscale score should be transformed onto a scale from 0 to 100 (a minimum score of 0 = no use of that process, a maximum score of 100 = full-use of the process). A higher use of a process is represented by scores above 50, while a lower use of a process by scores blow 50.	The S-Weight and the P-Weight questionnaires assess the relationship between stages and processes of change in weight-management. The S-Weight is able to detect five SOC (including the Preparation phase). The four-factor structure of the P-weight has been supported by principal component analysis (PCA) and confirmatory factor analysis (CFA). The P-Weight has good internal consistency (Cronbach’s alpha coefficients from 0.781 to 0.938 for the different subscales). The total scale has excellent internal consistency (total Cronbach’s alpha is 0.960) and adequate item-total correlations (Cronbach’s alpha from 0.322 to 0.865). It has satisfactory convergent validity with the other scales measuring concern with dieting (‘drive for thinness’ subscale of the EDI-3 and the ‘diet’ subscale of the EAT-40). The S-Weight and the P-Weight have been validated in both non-clinical (556 University students) and clinical samples (overweight and obese patients enrolled in a hospital-based weight-management program). Simple and short Likert-scale scoring; trained personnel are not required for administration for both tests. Scoring of both S-Weight and P-Weight can be carried out by hand, in around 15 min.	Subscales scores should be transformed onto a scale from 0 to 100 in order to be comparable with one another. To obtain the processes of change scores (from the P-Weight), across the SOC (from the S-Weight), more complex data analytic processes are needed, and the use of a statistical software is advisable. Unknown test–retest reliability. Clinical cut-offs of the P-Weight subscales are unknown.
University of Rhode Island Change Assessment Scale (URICA)	McConnaughy et al. (1983). SOC in psychotherapy: Measurement and sample profiles. Psychotherapy: Theory, Research, and Practice, 20, 368–375. McConnaughy et al. (1989). SOC in psychotherapy: a follow-up report. Psychotherapy, 26, 494–503.	32 items	Self-report measure assessing SOC on four subscales: Precontemplation, Contemplation, Action, and Maintenance. Responses on a 5-point Likert scale ranging from 1 (strong disagreement) to 5 (strong agreement). Item distribution is perfectly even: eight items for each of the four subscales. Subscales score range from a minimum of 1 to a maximum of 40. A continuous readiness to change (RTC) score is calculated by summing up scores on Contemplation, Action and Maintenance subscales and by subtracting the Precontemplation score. The total score ranges from a minimum of 1 to a maximum of 160. Scores below 80 reveal a total low readiness, while a high RTC is represented by a total score above 80.	Widely studied measure of RTC designed for an adult target population. It can be applied on a wide range of different problems (addictions, smoking cessation, and weight-control). Mainly useful for assessing patients in psychotherapy reporting on their specific problem in treatment. The four-factor structure of the test has been supported by principal component analysis (PCA) and structural equation modeling. In the clinical setting, the four subscales scores can be used to define behavior modifications according to the individual’s specific SOC. It has good internal consistency even in follow-up studies (coefficient alphas from 0.79 to 0.89 across subscales). Good construct validity is supported by factor and cluster analyses. It has good predictive validity, in foreseeing treatment attendance and weight-loss. Very easy to administer, it takes short time for completion and scoring (can be carried out by any staff by hand) and it does not require trained personnel.	It evaluates RTC on four SOC leaving out the Preparation one. It does not consider the processes of change. Weak test–retest reliability. Unclear structure and internal consistency on weight-control samples. The total score or the mean scale score may require cluster analysis and standardized scoring in large sample sizes. Clinical cut-offs in weight-management are unknown. Changes in pre–post design studies do not consider subscale shifts (into Action and Maintenance). Cluster profiling can reveal an overlap of subscales endorsement: a subscale score may be very close to the next, with potential underreporting of respondents who are ready to move to another stage.
Decisional Balance Inventory (DBI)	O’Connell and Velicer (1988). A decisional balance (DB) measure for weight loss. Intern. J. of Addictions, 23, 729–750 Rossi et al. (2001). Validation of DB and temptation measures for dietary fat reduction in a large school-based population of adolescents. Eating Behavior 2, 1–18.	20 Items	Self-report measure assessing decision-making for weight-control on two subscales: the pros and cons of losing weight. The test two-factor structure is supported by factor analyses and principal component analyses (PCA). Item distribution is perfectly even: 10 items per subscale. Responses on a 5-point Likert scale ranging from 1 (Not important at all), to 5 (Extremely important). The score of the Pros scale is calculated by summing up the scores obtained from all even numbered questions; the score of the cons scale is calculated by summing up the scores obtained from all odd numbered questions. Subscales score range from a minimum of 10 to a maximum of 50. A total DB score is gained by subtracting the cons score from that of the pros, with possible scores ranging from -40 to +40. Higher scores indicate more perceived cons than pros in managing weight.	It has very good internal consistency (Cronbach’s alpha coefficients of 0.91 and 0.84, respectively, for the pros and cons scales). It has fair construct validity on various health behavior problems (Prochaska et al., 1994; Redding et al., 2000). Completion brevity (5 min or less) Simple and short Likert-scale scoring; trained personnel are neither required for administration nor scoring.	It evaluates RTC only according to the pros and cons of decision-making. It does not consider the stages and the processes of change. No test–retest reliability. No data on factor structure or internal consistency on weight-control samples. Clinical cut-offs in weight-management are unknown. No published study has evaluated the psychometric properties of this measure in overweight or obese adults engaged in weight-loss treatments.

While the DBI only assesses decision making for weight-control by focusing on the pros and cons of losing weight (O’Connell and Velicer, 1988), it does not specifically concentrates on the stages nor on the processes of change individuals may go through when engaged in a weight-loss program. The theoretical framework of the DBI lies within the TTM model of change as decision-making processes are strictly related to the pros and cons depending on the individual’s stage of change (Di Clemente et al., 1991). Nonetheless, the tool has no specificity for what concerns the relationship between the SOC and individuals’ RTC in weight-management. Thus, the two-factor structure of the DBI only allows evaluating the respondent’s decision on whether or not to lose weight and it can be useful to gain a general total score on weight-management RTC (O’Connell and Velicer, 1988). Additionally, the questionnaire has only been validated on college students (O’Connell and Velicer, 1988), and in a large sample of adolescents undergoing a dietary fat reduction program (Rossi et al., 2001). Hence, research using the DBI in the clinical setting and on adults’ weight-control samples still needs to be carried out.

The URICA evaluates readiness for change in problem behaviors such as obesity, diet and weight management according to four SOC of the TTM model, namely Precontemplation, Contemplation, Action, and Maintenance (Prochaska and Velicer, 1997). The test global RTC score can be obtained by combining the scores from each of the four subscales. However, classifying participants into specific stages simply on the basis of the subscale scores can be difficult (Di Clemente and Hughes, 1990; Carney and Kivlahan, 1995). In other words, determining the numbers of cluster profiles may cause an overlap of subscales endorsement, suggesting that one’s stage designation may be somewhat uncertain. An individual’s score for one stage may be very close to the border of the next stage; for example, a precontemplation score might be very close to being a contemplation score. This could under report the number of respondents who are nearly ready to consider a move to make a meaningful change in their behavior (Blanchard et al., 2003). Moreover, the scale does not account for the processes of change which are important elements influencing behavior modification. Thus, the URICA represents only one way of evaluating motivation.

In addition, the questionnaire is not appropriate for a pre-post evaluation of treatment because some of the subscales change in direction and strength of endorsement as individuals abandon their dysfunctional behavior (Carney and Kivlahan, 1995; Di Clemente et al., 2004; Di Clemente, 2005). Thus, while results from the URICA provide adequate data by placing respondents into a representative category of a target-behavior and it is therefore a strong predictor at pre-treatment, this linear combination of subscales does not function well in post-treatment evaluations. This is due to the fact that time in treatment and recovery seems to change the relationships of the subscales outcomes (Carney and Kivlahan, 1995; Di Clemente et al., 2004; Di Clemente, 2005). Since the URICA is still being validated within the weight-management context, it is advisable to mainly utilize it for research purposes. Therefore, to date there have been no cut-off norms established to determine what constitutes high, medium or low on a particular stage in weight-control (McConnaughy et al., 1983, 1989).

Many motivational measures specifically developed for weight-management have not been adequately tested, while others may present some weaknesses (Palmeira et al., 2007). Amongst the ones selected and described by this paper, the S-Weight and P-Weight appear to be more useful and straightforward in evaluating RTC for weight-control, with the advantages regarding brevity and the constructs they analyze. Firstly, the S-Weight and P-Weight are able to measure both SOC that focus on when people change, as well as the processes of change which refer to how people change (Andrés et al., 2011). Secondly, unlike the DBI which concentrates on the DB formulated by the pros and cons of change, and the URICA which only measures four SOC, the S-Weight is able to detect five SOC, therefore adding the Preparation phase (Andrés et al., 2011) while the P-Weight provides information on the processes of change too.

In fact, the S-Weight and P-Weight are able to measure not only the corresponding motivational stage of the TTM model for weight-control, but they also focus on overt and covert activities that individuals engage in when they attempt to manage their weight, represented by the processes of change (Di Clemente et al., 1991). In fact, these are powerful predictors of behavior change and they can give crucial information on weight-management interventions efficacy (Prochaska et al., 1992b). Hence, assessing the processes of change can reveal how the patient is tackling his or her weight problem while the SOC classification complements the motivational evaluation by identifying when behavior change occurs. For such reasons, the use of the S-Weight and P-Weight to assess RTC in weight-management appears to be more resourceful than that of the URICA and the DBI. Finally, it must be considered that contrarily to the URICA and the DBI, the the S-Weight and P-Weight have been clinically validated in a sample of overweight and obese patients enrolled in a hospital-based weight-loss treatment.

The stage of change construct of the TTM model can facilitate intervention-tailoring by matching specific treatment strategies to individuals’ motivation and RTC (Jordan and Nigg, 2002). In other words, a stage-matched intervention for at-risk participants such as overweight and obese individuals can contribute to increase the chances of becoming more physically active and to decrease dietary fat intake, compared to a non-stage matched intervention (Steptoe et al., 2001). Promptly identifying stages and processes of change in weight-management can therefore help clinicians and other professionals developing a more suitable weight-specific intervention in order to determine which behaviors an individual should target for change, at various points during treatment. In this respect, the S-Weight and P-Weight seem to be more advantageous compared to the DBI and the URICA, considering their specificity and their psychometric properties in obese and overweight hospitalized in-patients.

## Limitations of the Review

This review presents some relevant limitations as the selection of the three suggested set of instruments entirely refers to the specific motivational framework proposed by Prochaska and Di Clemente’s RTC and SOC concepts of the TTM (Prochaska et al., 1994, 2008; Prochaska and Velicer, 1997; Prochaska and Norcross, 2006). Therefore, other important instruments which are often used in the clinical practice to evaluate weight-management motivation in overweight or obese individuals may have been left out. For example, the paper does not take into account two well-established instruments in this field such as the Treatment Self-regulation Questionnaire (TSRQ; Levesque et al., 2007) and the Weight-Efficacy Lifestyle Questionnaire Short Form (WEL-SF; Ames et al., 2012).

The TSRQ examines autonomous and controlled motivation on entering a weight-loss program (baseline) and continuing the program participation (follow-up). This questionnaire evaluates the motivational level of people engaged in weight-management treatments, and the reasons why they enter, follow and continue weight-loss programs. Thus, the scale investigates on the degree to which a person’s motivation for their health behavior is relatively autonomous (Levesque et al., 2007). The WEL-SF was developed to explore a sense of self-efficacy for dieting. The scale evaluates participants’ confidence in their ability to lose weight on five main dimensions: Negative Emotions, Availability, Social Pressure, Physical Discomfort, and Positive Activities. The TSRQ and the WEL-SF are often used within the primary care setting in order to identify specific RTC in weight-management, within overweight and obese individuals engaged in weight-loss treatment interventions (Ames et al., 2012).

## Conclusion

The TTM conceptualizes RTC across a wide range of behaviors, defining the relationships between stages and processes of change, DB, self-efficacy and relapse (Prochaska and Di Clemente, 1986). The use of the TTM constructs can enhance motivational techniques and encourage weight-loss by favoring adherence to weight-loss and weight management programs, with positive results (Wilson and Schlam, 2004; Ryan et al., 2011; Pietrabissa et al., 2012). In fact, the SOC can provide fundamental information toward the creation of well-tailored interventions according to the patient’s specific motivational level in weight-management (Prochaska et al., 1992b; Norcross et al., 2011). Questionnaires evaluating TTM constructs in the context of weight-management are usually adapted from instruments designed to measure motivation in addictive disorders (Greene and Rossi, 1998; Marshall and Biddle, 2001). In weight management, the assessment of stages and processes of change is usually carried out through the assessment of dietary fat reduction and exercise, separately. Using specific measures of both these behaviors at once, can contribute to offer a reliable and efficient assessment of weight-management motivation (Horwath, 1999).

Assessing motivation and RTC levels in weight-management can have an important impact on the outcome of efficient weight-control treatment interventions. By promptly identifying psychological obstacles or resistance to change in overweight or obese individuals engaged in a weight-loss treatment, could dramatically favor positive effects. Indeed, specific weight-loss interventions could be tailored according to certain behaviors individual should achieve in order to change, at different stages during treatment. It is fundamental to determine the motivational level of overweight or obese individuals enrolled in specific weight-loss programs, especially considering that most of them later go back to their original weight in three to 5 years after treatment (Castelnuovo et al., 2010).

In this respect, the URICA, the DBI and the S-Weight and P-Weight questionnaires are specifically able to measure RTC toward weight-control, according to the TTM model. Though all questionnaires appear to be all advantageous given their short-time completion and scoring and for the fact that no special training is required for administration, the S-Weight and P-Weight seem to be more efficient. Unlike the URICA and the DBI, the S-Weight and P-Weight specifically focus on RTC for weight-control by evaluating both stages and processes of change. These are two interrelated dimensions for the adequate assessment of behavioral modification. Moreover, the S-Weight and P-Weight have been validated in an overweight and obese in-patients sample, contrarily to the URICA and the DBI.

Thus, the S-Weight and P-Weight seem to provide a more adequate assessment of RTC in weight-control in-patients compared to the URICA and the DBI. They can therefore be of great help to clinicians and professionals who wish to provide patients with the best and most suitable weight-loss intervention. In fact, the S-Weight and P-Weight seem to be sound instruments in appraising RTC in weight-management, by giving a clear picture of which SOC individuals are in, and of the processes of change they are using. This information could favor the decrease or minimization of resistant and ambivalent behavior toward change. All in all, among the three sets of questionnaire analyzed in this work, the S-Weight and P-Weight are the most reliable and beneficial tools in assessing RTC in individuals engaged in weight-loss programs.

## Conflict of Interest Statement

The authors declare that the research was conducted in the absence of any commercial or financial relationships that could be construed as a potential conflict of interest.
